# Exosome-like vesicles in uterine aspirates: a comparison of ultracentrifugation-based isolation protocols

**DOI:** 10.1186/s12967-016-0935-4

**Published:** 2016-06-18

**Authors:** Irene Campoy, Lucia Lanau, Tatiana Altadill, Tamara Sequeiros, Silvia Cabrera, Montserrat Cubo-Abert, Assumpción Pérez-Benavente, Angel Garcia, Salvador Borrós, Anna Santamaria, Jordi Ponce, Xavier Matias-Guiu, Jaume Reventós, Antonio Gil-Moreno, Marina Rigau, Eva Colas

**Affiliations:** Biomedical Research Group in Gynecology, Vall Hebron Institute of Research (VHIR), Universitat Autònoma de Barcelona, Barcelona, Spain; Biomedical Research Group in Urology, Vall Hebron Institute of Research (VHIR), Universitat Autònoma de Barcelona, Barcelona, Spain; Department of Gynecology, Vall Hebron University Hospital, Barcelona, Spain; Pathology Department, Vall Hebron University Hospital, Barcelona, Spain; Grup d’Enginyeria de Materials (GEMAT), Institut Químic de Sarrià, Universitat Ramon Llull, Barcelona, Spain; Department of Gynecology, Bellvitge Teaching Hospital, Barcelona, Spain; Department of Pathology and Molecular Genetics/Oncologic Pathology Group, University Hospital Arnau de Vilanova, University of Lleida, IRBLleida, Lleida, Spain; Bellvitge Biomedical Research Institute (IDIBELL), Hospitalet de Llobregat, Barcelona, Spain; Basic Sciences Department, International University of Catalonia, Barcelona, Spain

**Keywords:** Biomarker, Endometrial biopsy, Exosomes, Exosomes isolation protocols, Exosome-like vesicles, Extracellular vesicles, Gynecological disorders, Microvesicles, RNA, Uterine aspirates

## Abstract

**Background:**

Uterine aspirates are used in the diagnostic process of endometrial disorders, yet further applications could emerge if its complex milieu was simplified. Exosome-like vesicles isolated from uterine aspirates could become an attractive source of biomarkers, but there is a need to standardize isolation protocols. The objective of the study was to determine whether exosome-like vesicles exist in the fluid fraction of uterine aspirates and to compare protocols for their isolation, characterization, and analysis.

**Methods:**

We collected uterine aspirates from 39 pre-menopausal women suffering from benign gynecological diseases. The fluid fraction of 27 of those aspirates were pooled and split into equal volumes to evaluate three differential centrifugation-based procedures: (1) a standard protocol, (2) a filtration protocol, and (3) a sucrose cushion protocol. Characterization of isolated vesicles was assessed by electron microscopy, nanoparticle tracking analysis and immunoblot. Specifically for RNA material, we evaluate the effect of sonication and RNase A treatment at different steps of the protocol. We finally confirmed the efficiency of the selected methods in non-pooled samples.

**Results:**

All protocols were useful to isolate exosome-like vesicles. However, the Standard procedure was the best performing protocol to isolate exosome-like vesicles from uterine aspirates: nanoparticle tracking analysis revealed a higher concentration of vesicles with a mode of 135 ± 5 nm, and immunoblot showed a higher expression of exosome-related markers (CD9, CD63, and CD81) thus verifying an enrichment in this type of vesicles. RNA contained in exosome-like vesicles was successfully extracted with no sonication treatment and exogenous nucleic acids digestion with RNaseA, allowing the analysis of the specific inner cargo by Real-Time qPCR.

**Conclusion:**

We confirmed the existence of exosome-like vesicles in the fluid fraction of uterine aspirates. They were successfully isolated by differential centrifugation giving sufficient proteomic and transcriptomic material for further analyses. The Standard protocol was the best performing procedure since the other two tested protocols did not ameliorate neither yield nor purity of exosome-like vesicles. This study contributes to establishing the basis for future comparative studies to foster the field of biomarker research in gynecology.

**Electronic supplementary material:**

The online version of this article (doi:10.1186/s12967-016-0935-4) contains supplementary material, which is available to authorized users.

## Background

Uterine aspirates (UAs), which are endometrial biopsies obtained by aspiration, are considered a very complex biological sample that highly represents the uterine cavity milieu. It combines the components of the uterine fluid (secretions from the luminal epithelium and glands, proteins selectively transferred from blood, and likely contributors from tubal fluid) with a cellular fraction (endometrial and blood cells) [1]. Thanks to its location and composition, UAs reflect cytological and molecular alterations present in tissues from the female genital tract [2, 3]. Therefore, this sample is currently used for histopathological examination, performed after the transvaginal ultrasonography, to diagnose endometrial disorders [4, 5]. Biomedical research on UAs is limited. However, although few molecular studies have been performed, those have significantly contributed to improving sensitivity and negative predictive value of UAs as a diagnostic tool for endometrial cancer [2, 3, 6]. To expand research in the field of biomarker discovery for gynecological pathologies using UAs, exosome-like vesicles (EVs) arise as a promising source of biomarkers.

EVs are 20–200 nm round membrane vesicles [7–9] released by multivesicular bodies fusing with the cell membrane [10, 11]. These lipid bilayer entities bear well-protected proteins, lipids, and RNAs, mediating intercellular communication between different cell types [12–15]. Specific sorted information is horizontally transferred from the cells of origin to other cells, influencing the recipient cell functions [16]. EVs are constantly released by cells in circulation or proximal body fluids, and therefore, they have been described in blood [17], urine [18], saliva, and breast milk [19], among other body fluids. Differently, microvesicles (MVs) are 100–1000 nm vesicles originated by budding/shedding of the plasma membrane [20]; their size range overlaps partially with that of EVs, hence hindering a complete size-discrimination between these two populations of extracellular vesicles [21, 22]. The features of EVs have fostered biomarker research in many diseases [16, 23–25]. However, a major bottleneck when performing EVs-based studies is the lack of standardization for already challenging techniques to isolate and characterize EVs. Since EVs reflect the status of the originating cell [23], we propose the UAs’ fluid fraction as a promising source of EVs to find molecules that could improve the diagnosis, prognosis, and treatment of gynecological alterations. Here, we aim to determine whether EVs exist in UAs and to compare protocols for their isolation, characterization, and further RNA analysis.

## Methods

### Sample collection and processing

A total of 39 pre-menopausal patients with benign gynecological diseases or healthy donors who came to the Unit of Gynecology at Vall Hebron University Hospital were recruited following the ethically approved protocol for this study (approval number: PR_AMI_50-2012). All patients signed the informed consent. A description of the clinic-pathological features of all participating patients is detailed in Additional file 1: Table S1. An inclusion criterion was pre-menopause. Women who had been treated previously for gynecological pelvic cancer, as well as patients positive for the human immunodeficiency virus and/or the hepatitis virus were excluded.

UAs were obtained by aspiration with a Cornier Pipelle (Gynetics Medical Products). Samples were placed in 1.5 mL tubes and kept on ice through all the processing which included addition of phosphate buffered saline (PBS) in a 1:1 ratio (v/v), gently pipetting of the sample and centrifugation at 2500*g* (4 °C) in a F45-30-11 rotor (Eppendorf Microcentrifuge 5417R) for 20 min to remove the cellular fraction. The remaining fluid fraction of the UA, from now on referred to as Supernatant (SN) fraction, was then aliquoted and frozen at −80 °C until needed. To compare isolation protocols, a pool of 27 SNs (samples 1–27; Additional file 1: Table S1) were mixed and divided into 20 aliquots containing 445 µL.

### Isolation of EVs

**Fig. 1:**
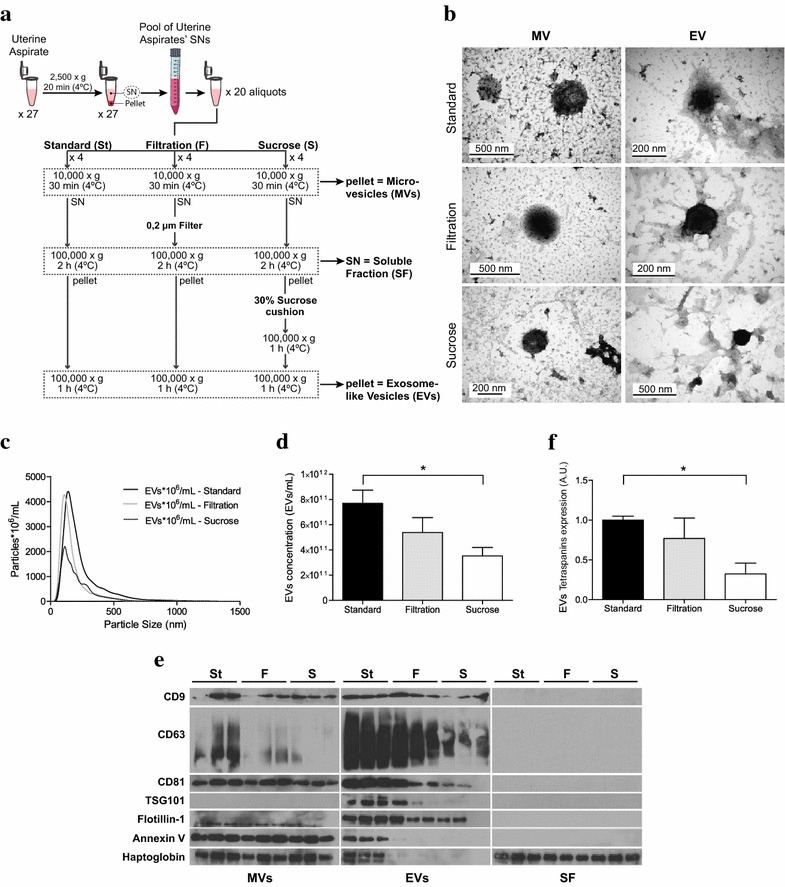
Optimization of EVs isolation from uterine aspirates. **a** Schematic representation of the three protocols of EVs isolation, namely Standard (St), Filtration (F), and Sucrose (S) protocols. **b** Electron microscopy images of negatively stained EVs and MVs. **c** Size distribution of isolated EVs measured by NTA. **d** EVs concentration measured by NTA. **e** Immunoblot of isolated MVs, EVs and SF proteins (done in triplicates) against EVs/MVs markers and Haptoglobin. **f** Relative tetraspanins expression of EVs. The average of tetraspanins (CD9, CD63 and CD81) expression of each protocol was normalized to the Standard in order to have a relative measurement of EVs purity

Protocols described in sections “I”, “II”, and “III” (Fig. 1a) were performed in quadruplicates to optimize EVs isolation. To optimize miRNA/mRNA extraction, modifications of the Standard protocol were tested in duplicates—section “IV” (Fig. 3a).

#### Standard protocol

EVs were obtained from the SNs of UAs by differential centrifugation, following a modification of a previously described EVs isolation protocol by Thery et al. [26]. Briefly, SNs were thawed and diluted in PBS to a final volume of 25 mL. A centrifugation step at 10,000*g* (4 °C) for 30 min was performed on a Thermo Scientific Heraeus MultifugeX3R Centrifuge (FiberLite rotor F15-8x-50c) to remove cell debris, macroparticles and apoptotic bodies. The resulting pellet enriched in MVs was resuspended in 50 µL of PBS and frozen at −80 °C. Then, the supernatant was transferred to ultracentrifuge tubes (Beckman Coulter) and filled with PBS to perform a first ultracentrifugation step at 100,000*g* (4 °C) for 2 h on a Thermo Scientific Sorvall WX UltraSeries Centrifuge with an AH-629 rotor. The supernatant of this second centrifugation was the soluble fraction and was frozen at −80 °C. This first pellet was resuspended in PBS and again centrifuged at 100,000*g* (4 °C) for 1 h. The final pellet enriched in EVs (possibly along with MVs and some remaining apoptotic bodies) was resuspended in 50 µL of PBS. Five microliters from MVs and EVs pellets were reserved at −80 °C for particle size distribution and quantification by nanoparticle tracking analysis (NTA) while the rest of the sample was frozen at −80 °C for protein extraction.

#### Filtration protocol

The Filtration protocol consisted in depleting the supernatant obtained after the 10,000*g* centrifugation of structures bigger than 200 nm using a sterile filter (Corning). The rest of the protocol remains the same as for the Standard.

#### Sucrose cushion protocol

A 30 % sucrose cushion (20 mM Tris–HCl, pH 7.4 in D_2_O)—density from 1.13–1.19 g/mL—was introduced to the Standard protocol following the first ultracentrifugation at 100,000*g*. The sucrose cushion was then centrifuged for 1 h at 100,000*g* (4 °C). EVs were recovered by poling the tube with a needle and were then washed with PBS for 1 h at 100,000*g* (4 °C). The final pellet was resuspended in 50 μL of PBS and the same fractions as in Section “I” were stored at −80 °C.

#### Experimental conditions for RNA extraction

Four experimental conditions (“A”, “B”, “C” and “D”) derived from the Standard protocol were tested, including sonication and RNase A treatment at different points, as shown in Fig. 3a. EVs were sonicated five cycles of 5 s at 100 Amplitude (Sartorius). For RNase A treatment, EVs were incubated with 500 µL of 0.1 mg/mL RNase A (Qiagen) for 1 h at 37 °C.

### Particle size distribution and quantification

NTA was performed using a NanoSight LM10 system (Malvern Instruments) equipped with a 405 nm laser and a Hamamatsu C11440 ORCA-Flash2.8 camera (Hamamatsu). Data was analyzed with the NTA software 2.3. Size and concentration of particles were determined by the following settings: camera level and detection threshold were set to maximum (15 or 16) and minimum (3–5), respectively; camera gain was set to 512; blur, minimum track length, and minimum expected size were set to “auto.” Readings were taken in triplicates during 60 s at 18.87 frames/s, at room temperature ranging from 23–25 °C.

### Electron microscopy

Isolated MVs and EVs were analyzed per duplicate by transmission electron microscopy (TEM). Vesicles were fixed in 50 µL of 4 % paraformaldehyde. Gold grids were incubated with samples for 1 min. After removing sample excess, negative staining was performed by incubation with uranyl acetate for 1 min. After washing, grids were dried overnight at room temperature. Samples were observed with a transmission electron microscope JEOL 1010 coupled to an Orius CCD camera (Gatan, Inc.), working at 80 kV with a tungsten filament.

### Protein extraction

Protein extraction of EVs and MVs was performed by adding RIPA buffer (40 mM Tris pH 8, 300 mM NaCl, 10 mM EDTA, 2 %Triton X-100, 1:100 protease inhibitors (#P8340 Sigma-Aldrich) in 1:1 ratio (v/v) to isolated vesicles and incubating at −20 °C overnight. Lysates were thawed on ice and sonicated five cycles of 5 s at amplitude 100 (Labsonic M, Sartorius Stedim Biotech) to ensure membrane disruption. Protein extraction of the soluble fraction collected after the 2 h ultracentrifugation step was performed by precipitation with 100 % stock solution of acetonitrile at a ratio of 1:5 (v/v) after incubation at −20 °C overnight, and sequential centrifugations at 14,000×*g* 4 °C for 30 and 15 min, respectively. Finally, the pellet was dried and resuspended in 500 µL of RIPA. Protein concentration was determined by Bio-Rad DC Protein Assay (Bio-Rad Laboratories) following manufacturer’s recommendations.

### Immunoblot

Proteins were separated by 10 % SDS-PAGE and transferred to PVDF membranes. For blocking, membranes were soaked in 5 % non-fat dried milk in TBS-Tween20 (0.01 %). Proteins were immunodetected using primary antibodies. Then the membranes were washed and incubated with a secondary HRP-coupled antibody. Finally, HRP signal was revealed using the Immobilon Western Chemiluminescent HRP Substrate (ref. WBKLS0100; Merck Millipore). The intensity of the bands was densitometrically quantified using the Image J software (v. 1.45s).

Primary antibodies: mouse anti-CD9 (1:250; ref. 555370, BD Biosciences), mouse anti-CD63 (1:1000; ref. OP171, Calbiochem), mouse anti-CD81 (1:1000; ref. sc-166028, Santa Cruz), mouse anti-TSG101 (1:500; ref. Ab83, Abcam), mouse anti-Flotillin-1 (1:250; ref. 610821, BD Biosciences), rabbit anti-Annexin V (1:1000; ref. ab108321, Abcam) and mouse anti-Haptoglobin (1:1000; ref. ab13429, Abcam). Secondary antibodies: rabbit anti-mouse Immunoglobulins/HRP, 1:2000, ref. P0260, Dako; and goat anti-rabbit Immunoglobulins/HRP, 1:2000, ref. P0448, Dako. Bands’ intensity was quantified using the Image J software (v. 1.45s).

### Total RNA extraction

Total RNA, including miRNAs and other RNAs, was isolated using Qiazol and miRNeasy MiniKit (Qiagen) according to manufacturers’ protocol. DNase I treatment (Qiagen) was used. RNA from EVs and MVs was eluted with 20 µL of Nuclease-free water (Ambion) and stored at −80 °C until further analysis. In all RNA extractions performed, 25 nmol of synthetic nonhuman miRNA-39 from *Caenorhabditis elegans* (cel-mir-39, 5′-UCACCGGGUGUAAAUCAGCUUG-3′) was added to each Qiazol lysate as a spike-in control for normalization in quantitative Real-Time qPCR (RT-qPCR) analysis [27–29]. RNA concentration and integrity were determined by capillary electrophoresis using the Agilent RNA6000PicoKit on an Agilent2100 Bioanalyzer (AgilentTechnologies).

### Reverse transcription, pre-amplification, and RT-qPCR

For mRNA analysis, RNA was converted to cDNA using SuperScript III Reverse Transcriptase (Invitrogen). For miRNA analysis, RNA was reverse-transcribed using TaqMan MicroRNA Reverse Transcription Kit and miRNA-specific stem-loop primers. All cDNA was pre-amplified with TaqMan Preamp Master Mix Kit (Applied Biosystems) according to manufacturer’s instructions.

RT-qPCR was performed using TaqMan Universal MasterMix II, with UNG on an ABI7900 Real-Time PCR Systems with TaqMan probes against specific transcripts and miRNAs. Reactions were performed in triplicate, and only results with a standard deviation value <0.37 were accepted. Data analysis was done with Expression Suite Software v1.0; the same baseline and threshold were set for each plate to generate threshold cycle (Ct) values for all the targets in each sample. Threshold levels were set into the exponential phase of the RT-qPCR. Synthetic cel-mir-39 was used for data normalization since the same amount of the oligonucleotide was added to each sample before the addition of the lysis reagent for RNA isolation.

### Real-Time qPCR TaqMan probes

All probes were purchased from LifeTechnologies. TSG101 (Hs00173072), PDCD6IP-ALIX (Hs00183813_m1), CD24 (Hs02379687), MUC16-CA-125 (Hs01065189), MUC1 (Cf02626759_m1), 18S (4319413E), β-Actin (4333762T), GAPDH (Hs99999905_m1), miR-200b (002251), miR-200c (002300), miR-223 (002295), miR-141 (000463), miR-205 (000509), miR-17 (002308), miR-106a (002169), RNU48 (001006), RNU6B (001093), RNU44 (001094), U75 (001219), and miR-39 (000200).

### Statistical analyses

Statistical analyses were performed using GraphPad Prism 5 software. The Student’s t test was applied to compare means of EVs concentration, particle size distribution, and expression of tetraspanins and miRNAs. The Pearsons’ Rho test was used to analyze correlation. The probability of p < 0.05 was considered statistically significant.

## Results

### EVs are present in the fluid fraction of uterine aspirates and can be isolated by differential centrifugation-based protocols

A pool of 27 UAs’ fluid fractions was used to compare three EVs isolation protocols based on differential centrifugation—Standard, Filtration and Sucrose. In addition to EVs, we collected fractions corresponding to MVs and proteins from the soluble fraction to monitor differences in the enrichment in EVs. A schematic representation of the experimental work is depicted in Fig. 1a.

All three protocols permitted the isolation of EVs of the expected round cup shape [30], as observed by TEM (Fig. 1b). Both EVs and MVs appeared as well-defined bilayer vesicles but notably, the size of all EVs was smaller than that for MVs, especially in the case of Standard protocol. To further investigate EVs concentration and size distribution, samples were analyzed by NTA (Fig. 1c, d). The population of EVs isolated by the Standard and Filtration protocols followed a uniform size distribution with a unique peak corresponding to a mode of 135 ± 5 and 115 ± 3 nm, respectively. For the Sucrose protocol, the distribution was not uniform; the mode was 135 ± 42 nm but presented an additional peak around 300 nm, and a high standard deviation was observed indicating less reproducibility of this isolation protocol. Differently, all MVs preparations presented a heterogeneous distribution, and a lower concentration than that for EVs (Additional file 2: Figure S1).

A reduction in EVs concentration was seen as more steps were added to the isolation protocol; significant differences were observed between Standard and Sucrose protocols (p = 0.029), and the same tendency was observed when comparing the Standard and Filtration protocols (Fig. 1d). To evaluate the purity of EVs obtained from each isolation protocol, we performed an immunoblot loading equal amounts of protein, and demonstrated that the expression of the tetraspanins CD63, CD9, and CD81—all considered late endosomal markers enriched in EVs [22, 31, 32]—was significantly higher in the Standard compared to the Sucrose protocol (p = 0.001) (Fig. 1e, f). The same tendency was observed for TSG101, a known endosomal origin marker [33], and Flotillin-1, an element of the membrane lipid rafts [34, 35]. These two markers were practically undetectable in MVs preparations, indicating a different biogenesis of these vesicles. As expected, Annexin V, a marker of MVs [36], was highly expressed in all MVs preparations; however, it was also detected in EVs derived from the Standard protocol suggesting that the smallest MVs populations might have precipitated at 100,000*g* or that specificity of this marker is arguably. None of the MVs or EVs markers were detected in the soluble fraction, but haptoglobin—an abundant protein found in blood—was highly expressed (Fig. 1e).

Altogether, we demonstrated that all protocols were able to enrich the sample in EVs. Nevertheless, we selected the Standard protocol for further applications since it allowed to obtain a higher EVs concentration, while maintained higher EVs-related markers and better reproducibility than the other tested protocols.

**Fig. 2:**
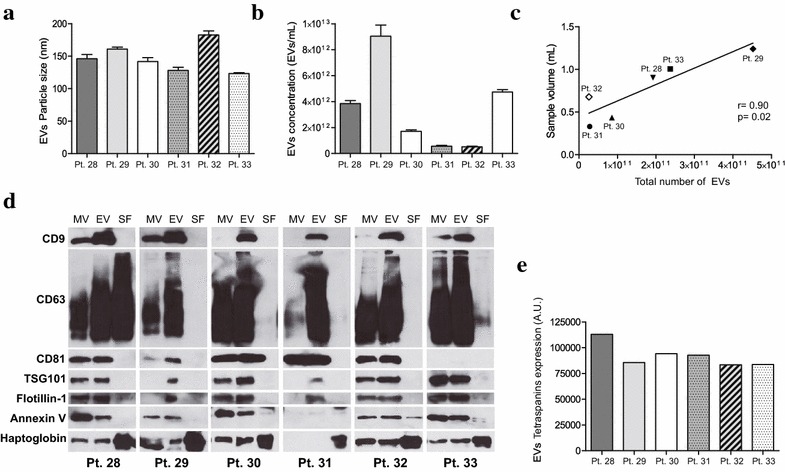
Characterization of EVs from individual uterine aspirates. **a** Particle size and **b** concentration of EVs was measured by NTA. **c** Correlation plot between total number of isolated EVs and starting volume of uterine aspirates’ fluid fraction. **d** Characterization of MVs, EVs and soluble fraction (SF) proteins by immunoblot against EVs/MVs markers and Haptoglobin. **e** Quantification of tetraspanins (CD9, CD63 and CD81) expression of EVs for each patient

To confirm that the enrichment in EVs following the Standard protocol holds when analyzing individual samples, we recovered EVs, MVs and soluble proteins from the fluid fraction of 6 non-pooled UAs (samples 28–33, Additional file 1: Table S1). Concomitant to our previous observations in the pooled analysis, we observed that all EVs preparations from individual UAs had a similar size distribution, presenting a mode of 120–160 nm (Fig. 2a). The particles concentration differed clearly between patients but a total number of isolated EVs significantly correlated with the initial volume of UAs fluid fraction (r = 0.90, p = 0.02) (Fig. 2b, c), but not with protein concentration (Additional file 3: Figure S2). On the other hand, no correlation was observed between a number of MVs and sample volume (Additional file 4: Figure S3). EVs markers were expressed in both EVs and MVs preparations from all patients (Fig. 2d, e). As seen previously, tetraspanins expression was higher in EVs than in MVs, indicating that we isolated a population of vesicles enriched in EVs. Altogether, these results indicate that the Standard protocol is suitable to obtain EVs from individual UAs.

### Optimization of EVs isolation protocols for RNA analysis

**Fig. 3:**
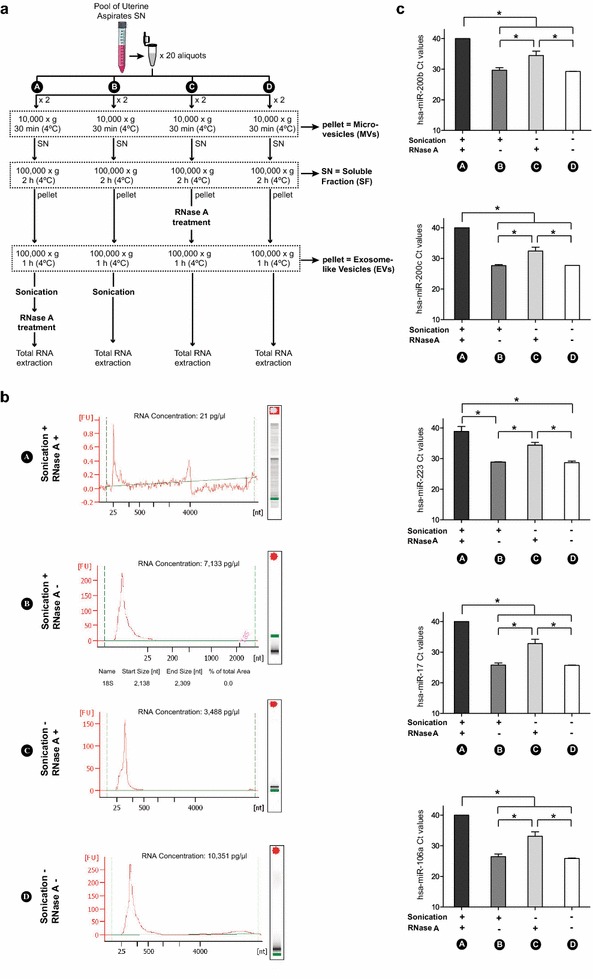
Optimization of EVs isolation from uterine aspirates for RNA analysis. **a** Schematic representation of the four conditions tested to isolate EVs from uterine aspirates in order to purify their RNA content. Modifications were introduced to the standard protocol of ultracentrifugation. Protocol “A”: Sonication prior to RNase A treatment was applied to isolated EVs. Protocol “B”: Sonication of isolated EVs. Protocol “C”: RNase A treatment of isolated EVs. Protocol “D”: No modifications were included. **b** Concentration and quality of RNA derived from each condition was analyzed with an Agilent Bioanalyzer. **c** Ct values of miR-200b, miR-200c, miR-223, miR-205, miR-17 and miR-106a are plotted for each Protocol

To further optimize the Standard protocol to extract RNA specifically contained in EVs, we evaluated the effect of sonication and RNase treatment, which enhances membrane disruption and promotes RNA release, and degrades RNA material, respectively (Fig. 3a). Concentration and quality of extracted RNA was determined by Bioanalyzer and RT-qPCR amplification of a set of miRNAs (miR-200b, miR-200c, miR-223, miR-17 and miR-106a), which were selected based on their expression in the female genital tract and their reported existence in EVs [22, 37–40]. Protocol “A”, which includes sonication before RNase A treatment, was used as negative control. This condition confirmed the successful breakage of EVs due to sonication and the successful degradation of RNA due to RNase A treatment, not obtaining RNA content nor amplification of any of the tested miRNAs (Fig. 3b, c). Protocols “B” and “D” did not deplete external RNA since RNase A was not applied; both protocols resulted in small size RNA profiles, yielding the highest amount of total RNA (Fig. 3b) and the highest expression of miRNAs (Fig. 3c). Importantly, the introduction of a sonication step did not report any advantage to enhance the release of EVs RNA content; conversely, we observed smaller RNA fragments, which are susceptible of RNA damage. In protocol “C”, RNase A was able to degrade 52 and 66 % of total RNA from treatment “B” and “D” respectively, suggesting that RNase A treatment is necessary to clean up the external nucleic acids that bind to EVs surface. This protocol (no sonication but RNase A treatment) was selected as the most appropriate to analyze RNA specifically contained within EVs.

**Fig. 4:**
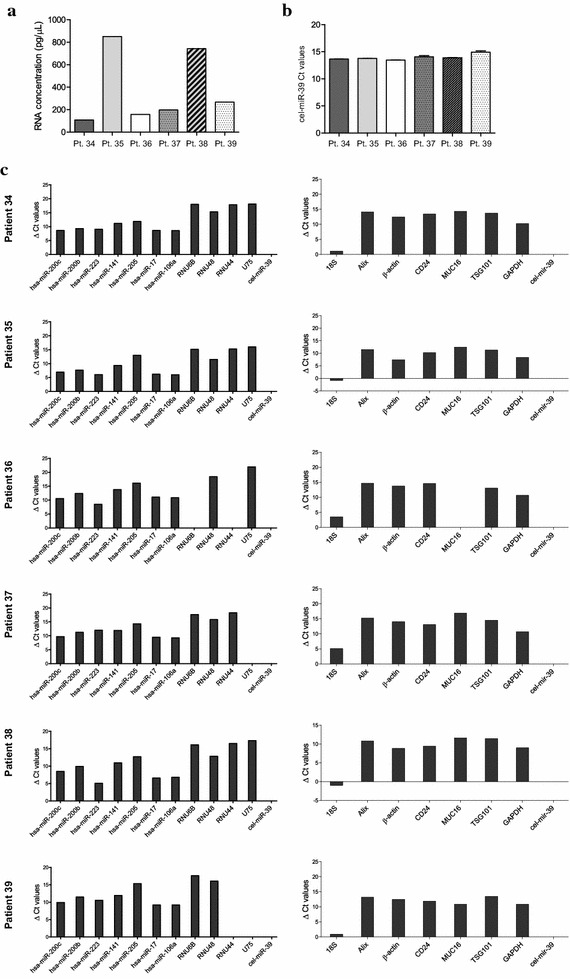
Characterization of EVs RNA content from individual uterine aspirates. **a** RNA concentration determined by Agilent Bioanalyzer. **b** Ct values of cel-miR-39 are plotted. **c** Expression of a set of miRNAs (*left*) and mRNAs (*right*) are ploted for each patient. Ct values were obtained by RT-qPCR and were normalized using miR-39 expression as a spike-in control

Next, we confirmed the efficiency of the selected protocol for RNA analysis in 6 additional non-pooled samples (Samples 34–39; Additional file 1: Table S1). Isolated EVs were similar in size, presenting a mode of 120–170 nm and contained RNA fragments from 25–300 nucleotides (Additional file 5: Figure S4). RNA concentration ranged from 108–851 pg/µL (Fig. 4a), which was sufficient to perform expression analysis by RT-qPCR. Sample volume, the total number of EVs and RNA concentration significantly correlated (Additional file 6: Figure S5A–C). Before RNA extraction, we added cel-miR-39 as a spike-in control for data normalization purposes. As seen in Fig. 4b, its expression did not vary across different samples, indicating similar RNA extraction efficiency. Afterward, we tested the amplification of a set of 11 miRNAs and 8 mRNAs by RT-qPCR, all of them previously reported in EVs [41, 42] (Fig. 4c). Delta Ct (dCt) values were relativized to the cel-miR-39 expression in each sample. We observed that 18S RNA presented the highest expression. Alix, TSG101, GAPDH and β-actin, EVs-related markers, along with MUC16 [43] and CD24 [44–46], proteins related to some gynecological alterations, were detected at RNA level. In addition to those tested for the optimization, we also analyzed the expression of other female genital tract-related miRNAs [38], miR-141 and miR-205, plus a set of tissue endogenous miRNAs [47], RNU6B, RNU48, RNU44, and U75. Expression of miR-200b, miR-200c, miR-223, miR-17, and miR-106a was higher compared to miR-205 and miR-141. Interestingly, RNU6B, RNU48, RNU44, and U75 were expressed at very low level in EVs. Detection of female genital tract associated RNA in EVs from UAs supported the idea that EVs cargo might reflect the cell status and/or its origin. However, this study was designed to confirm the appropriate extraction of RNA material to pursue further RNA analysis on EVs isolated from UAs, and is not intended to draw any disease-specific conclusion.

## Discussion

Here, we demonstrated that EVs exist in the fluid fraction of UAs by comparing three protocols of isolation, all of them based on ultracentrifugation, as this has been the method of choice for concentrating and isolating EVs in several body fluids [48]. Moreover, we carried out an extensive characterization describing their morphology, size and enrichment in well-known EV markers. When comparing the Standard, Filtration, and Sucrose protocols, we observed that all of them were capable of isolating EVs; but in particular, the Standard protocol permitted not only a higher recovery of EVs, but also a higher enrichment in tetraspanins. Furthermore, this protocol was the simplest, most reproducible and less costly protocol investigated here.

Many studies did not consider whether identified RNAs were contained in EVs or adhered externally to their outer membrane and, consequently, co-precipitated with EVs during the isolation protocol [49–51]. Here, we also established the optimal conditions to extract EVs RNA content treating isolated vesicles with RNase A and not applying sonication. Even though sonication was applied to successfully disrupt EVs membrane to improve protein yield [52], when this step was performed for RNA extraction, far from obtaining higher RNA concentration, we detected fragmentation and degradation. Thus, sonication of EVs is not appropriate for RNA studies; lysis reagent is efficient enough to break EVs membranes. Noteworthy, treatment with RNAse A was critical to eliminate the exogenous material while preserving the inner genomic content. We found that more than half of the RNA isolated from EVs preparations was exogenous. This abundant contamination should be considered, and if possible depleted when conducting transcriptomic studies. In line with this, a treatment to clean up EVs membranes from extraneous adhered proteins could have been tested. Trypsin is often used to break protein interactions; this property could be applied to analyze those proteins specifically contained in EVs. However, considering that the main EVs markers and possibly other proteins of interest are transmembrane structures, this digestion could affect the extracellular domains compromising protein structure, function and interaction with other proteins.

A wide range of different uterine specimens collected by various procedures is described in the literature [53–57]. Concomitant with our observation that female genital tract RNAs are detected in EVs from UAs, Vilella et al. proved that EVs isolated from endometrial fluids are certainly secreted by the endometrial epithelium cells, and consequently, their content may reflect the physiologic state of the uterine cavity. Importantly, these findings promote the use of EVs in UAs to search for those alterations that may originate from anomalous cells in the female genital tract, as the same rationale has been performed in other body fluids, such as bronchoalveolar lavage fluid of asthmatic patients [58] and urine of prostate cancer patients [59].

## Conclusions

We confirmed the existence of exosome-like vesicles in the fluid fraction of uterine aspirates. They were successfully isolated by differential centrifugation giving sufficient proteomic and transcriptomic material for further analyses. The Standard protocol was the best performing procedure since the other two tested protocols did not ameliorate neither yield nor purity of exosome-like vesicles. Certainly, our study contributes to standardize protocols and opens the door to conduct reliable and reproducible comparative studies using EVs isolated from UAs to foster the field of biomarker research in gynecology shortly.

## Additional files

10.1186/s12967-016-0935-4 Clinical and pathological features of patients. Age, diagnosis and starting volume of uterine aspirates’ fluid fraction are detailed. Samples 1-27 were pooled to compare EVs isolation protocols; samples 28-33 were individually used for EVs characterization by NTA and immunoblot; and samples 34-39, for the analysis of RNA content.
10.1186/s12967-016-0935-4 Size distribution of isolated MVs, all of them collected at the same point in each protocol.
10.1186/s12967-016-0935-4 Correlation plot between total number of isolated EVs and EVs protein concentration.
10.1186/s12967-016-0935-4 Correlation plot between total number of isolated MVs and starting volume of uterine aspirates’ fluid fraction.
10.1186/s12967-016-0935-4 (**A**) Correlation plot between total number of EVs and sample volume. (**B**) Correlation plot between total number of EVs and EVs RNA concentration. (**C**) Correlation plot between EVs RNA concentration and sample volume.
10.1186/s12967-016-0935-4 Concentration and quality of RNA derived from each individual uterine aspirate was analyzed with an Agilent Bioanalyzer.

## Abbreviations

UAs: uterine aspirates
EVs: exosome-like vesicles
RNA: ribonucleic acid
MVs: microvesicles
PBS: phosphate buffered saline
SN: supernatant
miRNA: micro ribonucleic acid
mRNA: messenger ribonucleic acid
NTA: nanoparticle tracking analysis
RNAse: ribonuclease
TEM: transmission electron microscopy
EDTA: ethylenediaminetetraacetic acid
SDS-PAGE: sodium dodecyl sulfate polyacrylamide gel electrophoresis
PVDF: polyvinylidene fluoride
TBS: tris-Buffered Saline
HRP: horseradish peroxidase
DNase: deoxyribonuclease
qPCR: quantitative polymerase chain reaction
cDNA: complementary DNA
Ct: threshold cycle
dCt: delta threshold cycle
