# Supplementary material for: Exosome-like vesicles in uterine aspirates: a comparison of ultracentrifugation-based isolation protocols
Source: J Transl Med. 2016 Jun 18;14:180. doi: 10.1186/s12967-016-0935-4 (PMC4912787; doi:10.1186/s12967-016-0935-4)
Supplement: Supplementary file 1 — 10.1186/s12967-016-0935-4 Clinical and pathological features of patients. Age, diagnosis and starting volume of uterine aspirates’ fluid fraction are detailed. Samples 1-27 were pooled to compare EVs isolation protocols; samples 28-33 were individually used for EVs characterization by NTA and immunoblot; and samples 34-39, for the analysis of RNA content. [file 12967_2016_935_MOESM1_ESM.docx]

**Table S1**

| **Patient** | **Age** | **Diagnosis** | **Aspirate fluid fraction volume** | **Experiment** |
| --- | --- | --- | --- | --- |
| 1 | 22 | Mucinous cystadenoma | 600 μL | Pool |
| 2 | 30 | Endometrioma | 760 μL | Pool |
| 3 | 24 | Follicular cyst | 550 μL | Pool |
| 4 | 36 | Papillary-serous cystadenoma | 1000 μL | Pool |
| 5 | 39 | Endometrioma | 1000 μL | Pool |
| 6 | 40 | Endometrioma | 650 μL | Pool |
| 7 | 23 | Endometrioma | 300 μL | Pool |
| 8 | 38 | Mucinous cystadenoma | 500 μL | Pool |
| 9 | 29 | Ovarian fibroma | 600 μL | Pool |
| 10 | 37 | Endometrioma | 450 μL | Pool |
| 11 | 37 | Endometrioma + uterine leiomyoma | 250 μL | Pool |
| 12 | 33 | Tubo-ovarian abscess + Endometrioma | 120 μL | Pool |
| 13 | 44 | Serous cystadenoma | 600 μL | Pool |
| 14 | 40 | Endometrioma | 400 μL | Pool |
| 15 | 21 | Serous cystadenoma | 400 μL | Pool |
| 16 | 38 | Mucinous cystadenoma | 600 μL | Pool |
| 17 | 31 | Mucinous cystadenoma | 100 μL | Pool |
| 18 | 51 | Proliferative endometrium | 500 μL | Pool |
| 19 | 28 | Mid-secretory endometrium | 500 μL | Pool |
| 20 | 47 | Endometrial polyp | 1000 μL | Pool |
| 21 | 45 | Uterine leiomyoma | 500 μL | Pool |
| 22 | 38 | Uterine leiomyoma | 300 μL | Pool |
| 23 | 46 | Normal endocervical tissue | 600 μL | Pool |
| 24 | 50 | Atrophic endometrium | 350 μL | Pool |
| 25 | 42 | Endometrial polyp | 160 μL | Pool |
| 26 | 36 | Endometrial polyp | 400 μL | Pool |
| 27 | 45 | Endometrioma | 500 μL | Pool |
| 28 | 30 | Endometrioma | 904 μL | Protein |
| 29 | 36 | Papillary serous cystadenoma | 1240 μL | Protein |
| 30 | 53 | Uterine submucosal leiomyoma | 434 μL | Protein |
| 31 | 28 | Normal endocervical tissue | 330 μL | Protein |
| 32 | 47 | Endometrial polyp | 678 μL | Protein |
| 33 | 51 | Secretory endometrium | 1000 μL | Protein |
| 34 | 30 | Uterine leiomyoma + adenomyosis | 730 μL | RNA |
| 35 | 39 | Unilateral Hydrosalpinx | 880 μL | RNA |
| 36 | 24 | Serous cystadenoma | 760 μL | RNA |
| 37 | 33 | Secretory endometrium | 750 μL | RNA |
| 38 | 40 | Uterine leiomyoma | 790 μL | RNA |
| 39 | 40 | Uterine leiomyoma | 750 μL | RNA |

**TABLE LEGENDS**

**TABLE S1. Clinical and pathological features of patients.** Age, diagnosis and starting volume of uterine aspirates’ fluid fraction are detailed. Samples 1-27 were pooled to compare EVs isolation protocols; samples 28-33 were individually used for EVs characterization by NTA and immunoblot; and samples 34-39, for the analysis of RNA content.
