# Supplementary figures and images for: Exosome-like vesicles in uterine aspirates: a comparison of ultracentrifugation-based isolation protocols
Source: J Transl Med. 2016 Jun 18;14:180. doi: 10.1186/s12967-016-0935-4 (PMC4912787; doi:10.1186/s12967-016-0935-4)

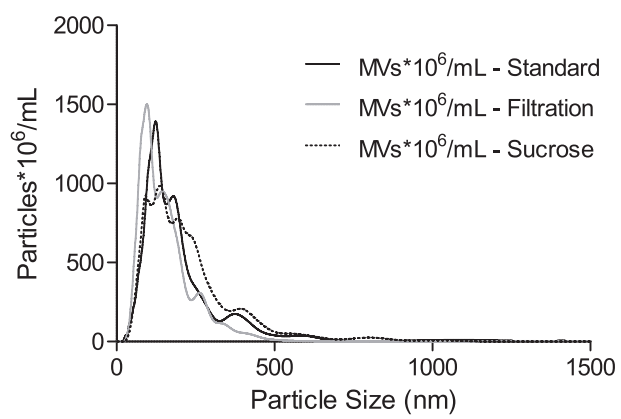

Supplement: Supplementary file 2 — 10.1186/s12967-016-0935-4 Size distribution of isolated MVs, all of them collected at the same point in each protocol. [file 12967_2016_935_MOESM2_ESM.pdf]

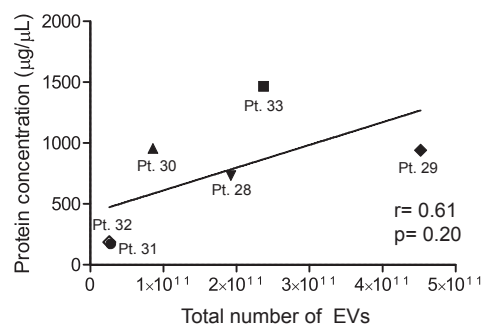

Supplement: Supplementary file 3 — 10.1186/s12967-016-0935-4 Correlation plot between total number of isolated EVs and EVs protein concentration. [file 12967_2016_935_MOESM3_ESM.pdf]

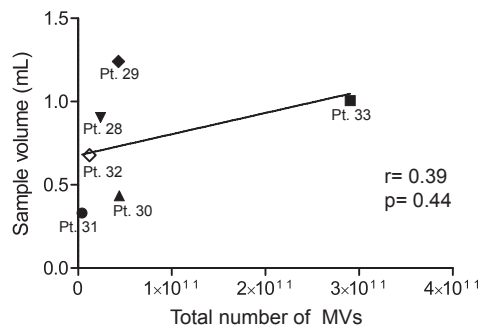

Supplement: Supplementary file 4 — 10.1186/s12967-016-0935-4 Correlation plot between total number of isolated MVs and starting volume of uterine aspirates’ fluid fraction. [file 12967_2016_935_MOESM4_ESM.pdf]

# EVs

Patient 34

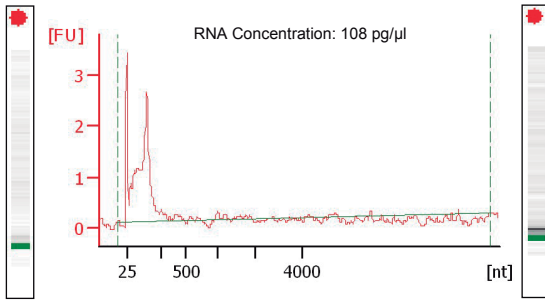

Patient 35

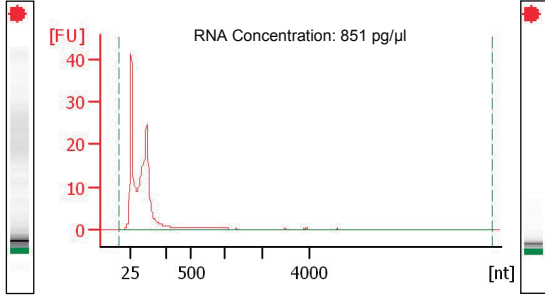

Patient 36

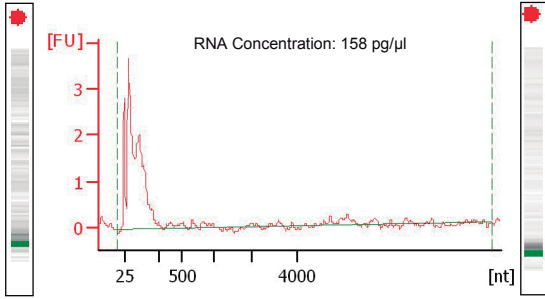

Patient 37

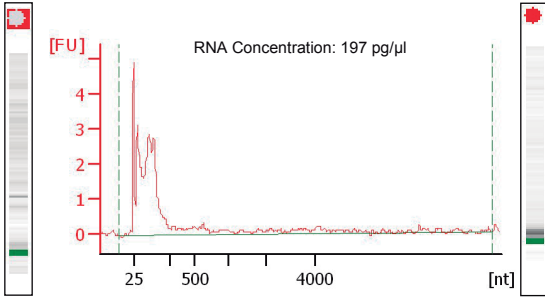

Patient 38

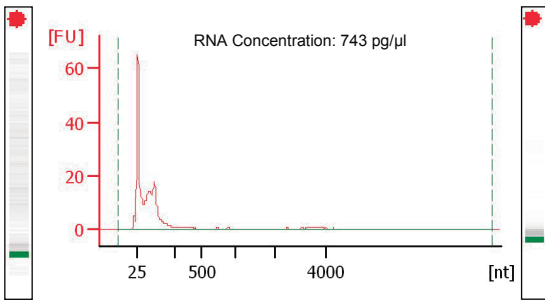

Patient 39

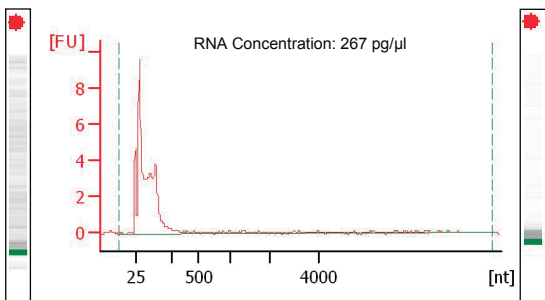

Supplement: Supplementary file 5 — 10.1186/s12967-016-0935-4 (A) Correlation plot between total number of EVs and sample volume. (B) Correlation plot between total number of EVs and EVs RNA concentration. (C) Correlation plot between EVs RNA concentration and sample volume. [file 12967_2016_935_MOESM5_ESM.pdf]

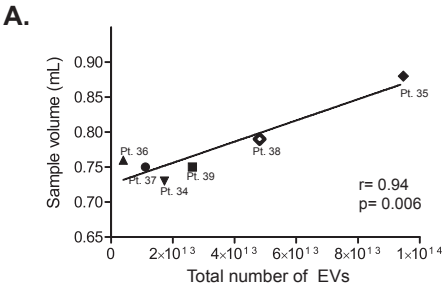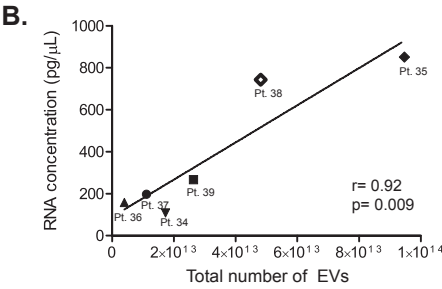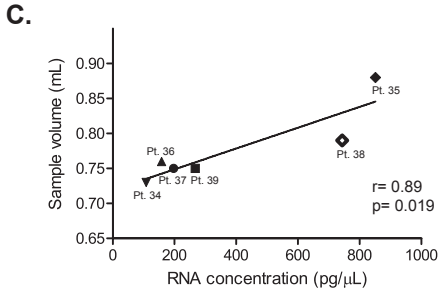

Supplement: Supplementary file 6 — 10.1186/s12967-016-0935-4 Concentration and quality of RNA derived from each individual uterine aspirate was analyzed with an Agilent Bioanalyzer. [file 12967_2016_935_MOESM6_ESM.pdf]
